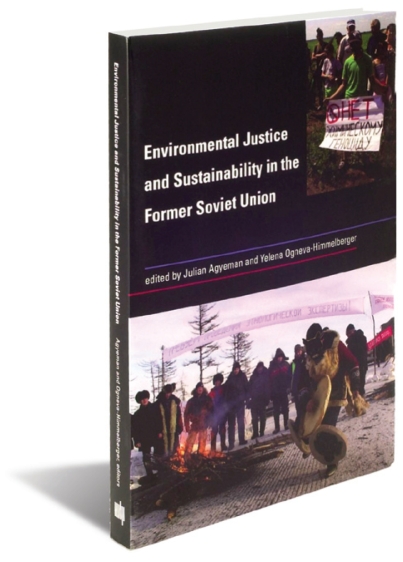# Environmental Justice and Sustainability in the Former Soviet Union

**Published:** 2010-09

**Authors:** Paul Josephson

**Affiliations:** *Paul Josephson teaches history at Colby College, Waterville, Maine, and is the author of several books on the history of science and technology in the former Soviet Union, most recently* Would Trotsky Wear a Bluetooth?

Widespread pollution in the former Soviet Union (FSU) led Murray Feshbach and Alfred Friendly Jr. to refer to the “ecocide” of Soviet citizens (*Ecocide in the USSR: Health and Nature Under Siege,* New York: Basic Books, 1992). Since the breakup of the Soviet Union, in many ways and in many places the environmental situation has improved. One of the reasons for this was plummeting industrial production, which led to the shuttering of inefficient factories; this occurred even though the newly independent states of the FSU lacked the resources, legal structures, political will, and/or public interest to deal with the environmental legacies of the Soviet Union. In addition, the number of nongovernmental organizations focused on environmental issues grew rapidly in the 1990s. How many of these organizations still operate and what their focus is remains unclear, especially since economic issues rather than environmental ones have diverted public attention.

In 10 chapters in *Environmental Justice and Sustainabilty in the Former Soviet Union*, specialists examine recent experiences with environmental justice in Russia, Sakhalin, Azerbaijan, Latvia, Kazakhstan, Sakha, Estonia, and Tajikistan. The authors’ goal is to answer to what extent increased popular environmental awareness and association activism drive public policy and planning in the former Soviet republics. They do so only to a very small extent; they conclude that emergent, separate environmental justice and environmentally sustainable development agendas have not joined into a single just agenda for sustainability or human security. The authors make no claim to be comprehensive in geography, analysis, and other issues, but have sought to begin a conversation and to establish a research agenda on the growing global awareness of justice, sustainable development, just sustainability, and human security.

I select a few of the chapters to give a sense of the important issues in this volume. Laura Henry points out how Russia has a legal foundation for environmental protection and indigenous people’s rights, but recentralization of power and the state’s unwillingness to implement laws have limited the effectiveness of environmental movements. Henry argues that Russian environmental actors and organizations focus on green issues, conservation, or environmental sustainability as opposed to just sustainability or human security. Henry explores why environmentalists in Russia today struggle to promote sustainability—her answer is growing political centralization and the fact that economic and social justice issues have become more crucial to the masses.

Many of the environmental issues in the FSU concern resource development—oil, gas, diamonds in Sakha, forests, and so on. Generally speaking, those who live near the resource suffer the immediate consequences of environmental degradation and see their standard of living drop, while individuals in the capitals or foreign investors profit. Kate Watters explores how one community strives to fight health effects of industrial oil development: Kazakhstan, which invited Western investment to develop those resources. Money flowed in for the benefit of the haves, while many who live close to the fields “live in dire poverty.” Jessica Graybill analyzes similar issues in Sakhalin.

Shannon O’Lear indicates that “environmental misfortune” in Azerbaijan because of oil exploitation is experienced by range of ethnic groups, not just one group at the expense of others. As usual, income differentials have risen, the percentage of people living in poverty has grown, and citizens unequally share the risks and benefits of the burgeoning oil industry. O’Lear’s survey data show that people who are better off perceive more significant pollution-related health effects than those poorer individuals who actually experience them.

Those who are not familiar with the environmental justice literature may find some of the discussion tending toward theoretical issues. I worry that the analysts in this volume overused the notion of “transitioning” states in the 1990s, and tied it teleologically to normative beliefs that all people would be better off in the FSU were the states to adopt some kind of Western democracy. This may be true, but there are tremendous assumptions here. Because the authors focus on the tension between “green” issues (those of environmental or sustainable development) and “brown” issues (those of environmental justice), I would have liked the editors to address this tension fully. Another assumption—again perhaps accurate—is that access to information and public participation are prerequisites for environmental justice. Passive voice abounds in several essays. Things are “thought to have grown,” “are transitioning,” “are largely occurring,” “are being raised,” with the result that the authors encounter challenges tying the analysis of discourses, world views, cognitive maps, and so on to concrete persons.

Political scientists, environmental studies specialists, and historians will find something of great interest in this volume. Anyone interested in contemporary environmental politics in the FSU should read this book. The introduction and the conclusion provide the framework for the case studies. Another strong point is a rich bibliography that accompanies each chapter.

## Figures and Tables

**Figure:**